# Low- and Negative-Pressure Hydrocephalus: New Report of Six Cases and Literature Review

**DOI:** 10.3390/jcm12124112

**Published:** 2023-06-18

**Authors:** Alicia Godoy Hurtado, Patrick Barstchi, Juan Francisco Brea Salvago, Rajab Al-Ghanem, Jose Manuel Galicia Bulnes, Osamah El Rubaidi

**Affiliations:** 1 Department of Neurosurgery, Jaén Neurotrauma Hospital, 23001 Jaen, Spain; patrick.barstchi.sspa@juntadeandalucia.es (P.B.); rajab.alghanem.sspa@juntadeandalucia.es (R.A.-G.); josem.galicia.sspa@juntadeandalucia.es (J.M.G.B.); osamah.abdullah.sspa@juntadeandalucia.es (O.E.R.); 2 Department of Neurocritical Care, Jaén Neurotrauma Hospital, 23001 Jaen, Spain; juanf.brea.sspa@juntadeandalucia.es

**Keywords:** low-pressure hydrocephalus, negative-pressure hydrocephalus, subzero drainage, external ventricular drainage, leptomeningeal glioneural tumor

## Abstract

Low- or very-low-pressure hydrocephalus is a serious and rare phenomenon, which is becoming better known since it was first described in 1994 by Pang and Altschuler. Forced drainage at negative pressures can, in most cases, restore the ventricles to their original size, thus achieving neurological recovery. We present six new cases that suffered this syndrome from 2015 to 2020: two of them after medulloblastoma surgery; a third one as a consequence of a severe head trauma that required bifrontal craniectomy; another one after craniopharyngioma surgery; a fifth one with leptomeningeal glioneuronal tumor; and, finally, a patient carrier a shunt for normotensive hydrocephalus diagnosed ten years before. At the moment of development of this condition, four of them had mid-low-pressure cerebrospinal fluid (CSF) shunts. Four patients required cerebrospinal fluid (CSF) drainage at negative pressures oscillating from zero to −15 mmHg by external ventricular drainage until ventricular size normalized, followed by the placement of a new definitive low-pressure shunt, one of them to the right atrium. The duration of drainage in negative pressures through external ventricular drainage (EVD) ranged from 10 to 40 days with concomitant intracranial pressure monitoring at the neurointensive care unit. Approximately 200 cases of this syndrome have been described in the literature. The causes are varied and superimposable to those of high-pressure hydrocephalus. Neurological impairment is due to ventricular size and not to pressure values. Subzero drainage is still the most commonly used method, but other treatments have been described, such as neck wrapping, ventriculostomy of the third ventricle, and lumbar blood patches when associated with lumbar puncture. Its pathophysiology is not clear, although it seems to involve changes in the permeability and viscoelasticity of the brain parenchyma together with an imbalance in CSF circulation in the craniospinal subarachnoid space.

## 1. Introduction

Low-pressure hydrocephalus has been described as a rare and severe syndrome in which there is impaired consciousness associated with ventricular dilatation without increased intracranial pressure [1,2,3].

The terms “normal pressure hydrocephalus”, classically “chronic Adult Hydrocephalus”, and “low pressure hydrocephalus” were used interchangeably until 1994 when Pang and Altschuler [3] described in detail the state of low-pressure hydrocephalus in a series of 12 patients. They had all been maintained previously on medium-pressure cerebrospinal fluid shunts and showed significant deterioration in the level of consciousness. This was not associated with either shunt dysfunction or increased intracranial pressure, but with the development of ventricular dilatation. All responded to drainage with subatmospheric pressures. Once the baseline ventricular size was recovered, patients received new shunts of variable pressures.

These authors defined this syndrome with the following criteria:

- The presence of previous shunts or drains;
- Ventriculomegaly despite low or normal pressures;
- Clinical and radiologic response to drainage at negative pressures;
- Exclusion of other causes such as system dysfunction.

Several hypotheses have been proposed as to its origin. The most widespread are the viscoelastic theory, due to a change in the elastic properties of the brain parenchyma, and the poroelastic theory.

There are still many questions to be answered about the pathophysiology of this syndrome. There is no consensus about the standard treatment, although even today, the most widespread modality is drainage at subatmospheric pressures until basal ventricular size is achieved, followed by the placement of definitive shunts [4].

A new series of six patients is provided here, with a review of the literature.

## 2. Materials and Methods

We identified those patients who met the criteria for low-pressure hydrocephalus prospectively at Jaén Hospital in the period 2015–2020. Some aspects of their evolution were completed retrospectively. For our review, we conducted a direct search on MEDLINE and Google Scholar using the terms “hydrocephalus” and “negative pressure” or “low-pressure”. Works concerning adult normotensive hydrocephalus were excluded.

The age range in the patients of our series was wide (9–80 years), and the underlying neurological pathology was equally varied. Four of them were minors: three had cranial tumor pathology (two with medulloblastomas and a third one with diffuse leptomeningeal glioneuronal tumor). The oldest patient was an 80-year-old woman, who had been diagnosed with chronic adult hydrocephalus ten years before. She had received a medium-pressure ventriculoperitoneal shunt (VPS) at diagnosis, to which she responded well.

The form of onset was similar, appearing in most cases (4 of the 6) as a somewhat abrupt deterioration (less than 24 h) of the level of consciousness in patients with VPS or recent cranial surgery, with a considerable increase in ventricular size in imaging tests (Evans index >0.3). Obstruction or dysfunction of the shunt was discarded and so was intracranial hypertension, measured through external ventricular drain (EVD) or intraparenchymal sensor.

All but one required intensive care unit management at some point during the episode. 

Once the low-pressure hydrocephalus condition was detected, and after unsuccessful surgical revision of the shunt system, four were treated with EVD, draining at a height of between 5 and 15 cm below the external auditory canal (EAC) for several days. Intermittent ICP measurements with closed drains ranged from −2 mmHg to zero. The other two subacute-impaired patients converted their ventriculoperitoneal shunt to a system connected alone to an antigravitational unit adjusted at zero mmHg as a free tube. During these changes of the shunt, some patients benefited from an intraparenchymal ICP measurement, which recorded finding pressures around zero with no pathological waves. 

In cases of EVD, once normal ventricular size and a good clinical condition had been achieved, the drainage height was progressively increased by 2 cm/day until positive pressures were reached. If the clinical condition was maintained without deterioration, surgery was programmed for the placement of a definitive shunt, with an adjustable very-low-pressure system or an antigravitational unit equally adjustable up to zero mmHg.

Follow-up lasted at least 1 year.

In all cases, we used ventricular catheters available on the market with the largest gauge (Codman, BACTISEAL^®^ with a diameter of 1.4 mm (Princeton, NJ, USA)).

## 3. Results

Bellow we develop the details of their evolution (Table 1).

### 3.1. Patient n. 1

11 year-old male patient was diagnosed with a posterior fossa lesion after consulting for vomiting and one month duration progressive headache and strabismus. Surgery was performed and complete resecction was achieved with histological resulting in desmoblastic medulloblastoma without meningeal involvement. 15 days after surgery the patient needed a CSF shunt due to clinical stagnation and increased ventricular size. Low pressure shunt was placed (Gav 5/35 Miethke^®^) with standardization of ventricular size, nevertheless the patient was affected by akinetic mutism and significant prostration. Radio and chemotherapy schedule was administered during following 2 months. The NMR after treatment showed considerable increase in venticular size so it was decided to replace shunt with Medtronic, Strata^®^ programmable system (Minneapolis, MN, USA) adjusted in 0.5. (1.5 mmHg). At this point the patient showed severe neurological impairment, without contact with the environment. In the absence of clinical or radiological response, multiple surgical revisions were carried out, including externalization of catheter several times.

Finally, the patient received an antigravitational device (Miethke, ProGav 2.0^®^ (Potsdam, Germany) associated with shunt and adjusted in 0 mmHg. Clinical and radiological improvement start one week after surgery. The patient could be discharged three weeks later, being able to recover oral feeding for the first time after the first surgery.

Overall hospitalization lasted 5 months since the first shunt dysfunction. Recovery continued until reaching a 80% Karnofsky Performance status 4 months later.

**Figure 1:**
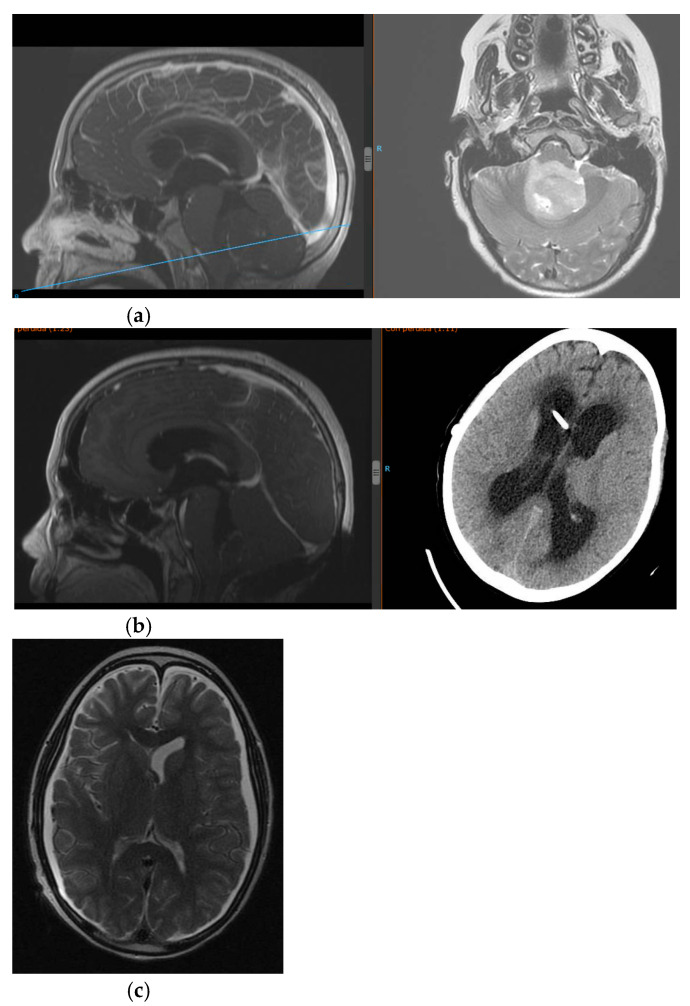
(**a**) NMR at admission showing fourth ventricle lesion and obstructive hydrocepahlus. (**b**) Postsurgical period with development of low-pressure hydrocephalus, needing several shunts revisions. (**c**) NMR one month after the placement of shunt asociated a PROSA^®^ device as “a free tube”.

Opening pressure reprogramming was needed to 4 mmHg due to asymptomatic subdural collections. 5 years after placement it maintains the same shunt system and opening pressure (Figure 1).

### 3.2. Patient n. 2

An 11-year-old male patient was admitted for progressive behavioral alteration and gait ataxia. He was diagnosed by imaging with a lesion in the fourth ventricle and meningeal involvement with secondary obstructive hydrocephalus. He urgently received an EVD for intracranial hypertension (IH). The patient underwent surgery two days later and was diagnosed with anaplastic giant cell medulloblastoma with meningeal involvement. The EVD was removed one week later, after verifying his tolerance to closed drainage for two days. Four days later, he suffered neurological worsening. Chemo- and radiotherapeutic treatment were started. The patient went into a state of akinetic mutism with severe impairment, no contact with the environment, incoercible vomiting, and prostration. Several digestive explorations were practiced with negative results.

**Figure 2:**
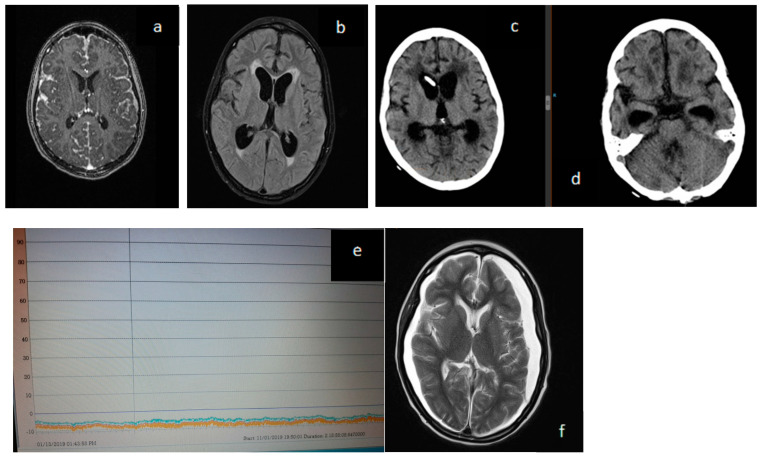
Images (**a**,**b**) show pre- and post-surgery for medulloblastoma with meningeal involvement. Images (**c**,**d**) correspond to the state of low-pressure hydrocephalus that was maintained for weeks. Image (**e**) continuous intraparenchymal ICP monitoring with externalized distal catheter and after its internalization as a “free tube”. This corresponds to CT images (**c**,**d**). Image (**f**) illustrates clinical improvement with marked hyper drainage and tumor response. The patient was carrying a MIETHKE proSA^®^ antigravitational unit adjusted at zero. Image (**f**) was taken 12 months later with the antigravitational unit adjusted at 40 mmHg.

In successive imaging tests, the tumoral meningeal involvement improved, but ventriculomegaly with ependymal transudation developed. A programmable ventriculoperitoneal shunt system was placed, which was ineffective despite the progressive lowering of the opening pressure. The patient was switched to an EVD by progressively lowering the drainage height below the EAC. Continuous recording of ICP with the intraparenchymal sensor during subzero drainage showed negative pressures, a flat recording with no pathological waves, and minimal amplitude between systolic and diastolic values, suggesting a very distensible or “compliant” brain (Figure 2) [5,6].

In order to continue with the oncological treatment, we decided to place a VPS associated with an antigravitational unit programmed to opening pressures of zero mmHg (Miethke proSA^®^) and monitoring with an intraparenchymal sensor and serial imaging (Figure 2). The patient continued his systemic treatment and recovery. It was not until 3 months after surgery that the neurological situation began to improve. This coincided with a striking decrease in ventricular size on imaging controls. Associated with this phenomenon, there were marked radiological signs of hyper drainage with bilateral subdural collections (Figure 2f) without any clinical repercussions. The opening pressure of the antigravitational unit was then raised to the maximum allowed (40 mmHg), showing a progressive reduction.

### 3.3. Patient n. 3

10 years old. Male. Initial admission for headache and coccygodynia. The initial examination revealed papillary oedema in the ocular fundus. The NMR showed normal ventricular size. He was initially diagnosed with idiopathic intracranial hypertension (IIH).

He improved with medium-pressure pediatric VPS (Aesculap pediGAV^®^ 9/29, Miethke).

**Figure 3:**
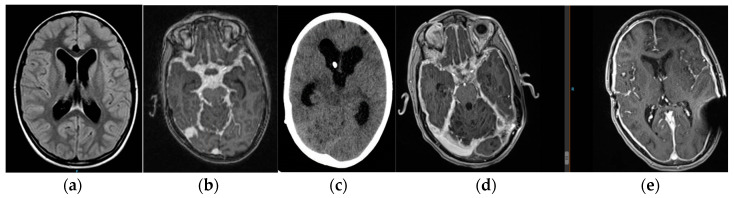
Image (**a**) shows condition prior to the placement of the first shunt system. Images (**b**,**c**) correspond to the moment when the patient was diagnosed with a leptomeningeal oncologic process and developed hydrocephalus, despite pressure reductions. Note the subarachnoid space occupied by tumor content. Images (**d**,**e**) show response to systemic treatment with visualization of the cisterns. The last CT even shows meningeal enhancement and laminar ventricles suggestive of hyper drainage.

One year later, he had generalized epileptic seizures that were difficult to control. NMR showed discrete meningeal enhancement, interpreted as hyper drainage, so he was switched to a programmable system (Codman Certas Plus^®^). Slow clinical deterioration continued. A year and a half after the onset of the symptoms, diffuse leptomeningeal glioneuronal tumor was diagnosed by biopsy. The disease progressed, requiring shunt revisions due to insufficiency despite reprogramming. He was switched to EVD with ICP monitoring and needed to remain at zero to stay awake and return to baseline ventricular size. Three weeks later, a programmable proGAV^®^ 2.0 system associated with a proSA^®^ antigravitational unit was placed, both at zero as a free tube. Oncological treatment continued with transitory decrease in the size of the lesions, which remained stable with a small ventricular system with slight signs of hyper drainage (Figure 3). Eventually, the patient died, three years after diagnosis.

### 3.4. Patient n. 4

80 years old. Female. The patient was diagnosed ten years earlier with chronic adult hydrocephalus in another center after consulting for gait disturbance. She received low-pressure VPS with an antigravitational unit with good response during this time. There was a progressive clinical worsening that required a valve change twice in one month. At the time of admission to our center, she was fitted with a system associated with an antigravitational unit (Aesculap GAV^®^ 0–30 mm H_2_O). The patient was in a coma and with tetraventricular dilatation (Evans 0.38). Surgical examination showed no dysfunction. She was switched to EVD with pressures of zero mmHg. Drainage 15 cm below the EAC was required initially to recover consciousness and baseline ventricular size.

**Figure 4:**
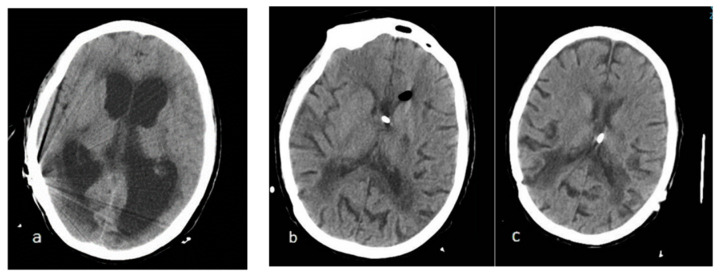
Image (**a**) shows patient in a coma at the time of the first surgical revision. Image (**b**) patient with EVD at pressures of −15 cm H_2_O with which she remains awake. Image (**c**) patient with the definitive shunt (Miethke M-Blue^®^ unit programmed at zero as a free tube) placed by neuronavigation due to the patient’s poor tolerance to ventricular dilatation. Note the transependymal edema that persists despite normalization of ventricular size.

Once basal size was recovered, the patient was unable to tolerate pressures above 2 cm of water, above which she would fall back into an arreactive coma. The whole time with forced negative pressure was 15 days. Once again, she received a system associated with an antigravitational unit, initially adjusted at zero (Figure 4).

The clinical evolution was very favorable, but 10 months later, a CT scan showed complete ventricular collapse, requiring a rise to 4 mmHg. The patient was asymptomatic.

### 3.5. Patient n. 5

42 years old. Male. History of retroperitoneal fibrosis.

The patient was diagnosed with craniopharyngioma after consulting for visual loss. He underwent surgery through a left pterional approach. The postoperative period was complicated with panhypopituitarism and septicemia of urinary origin. At 7° day after surgery he was diagnosed with hydrocephalus by CT. Clinically showed sleep tendency.

A programmable shunt system was placed (Codman CERTAS plus®) with progressive pressure drop at lowest level without clinically or radiologically improvement. Surgical revisions and catheter externalizations were carried out ineffectively. The patient responded to EVD draining at negative pressures which were reached progressively to 15 cm below de EAC, with daily debts around 300 mL of CSF. It took 25 days at intensive unit care to recover normal ventricular size associated with level of consciousness maintained. Then level of EVD was progressively raised until Monro level. On day 40 after the onset, he could receive a new shunt associated with an antigravitational device programmed at zero opening pressure with success (Miethke ProSA®). 7 days later the patient presented low level of consciousness again with a new ventricular dilatation. At this moment it was decided to place a concomitant catheter in contralateral ventricle and start again negative drainage. For 20 days he was maintained with the double catheter system. Progressive removal of the external catheter was possible as done previously.

**Figure 5:**
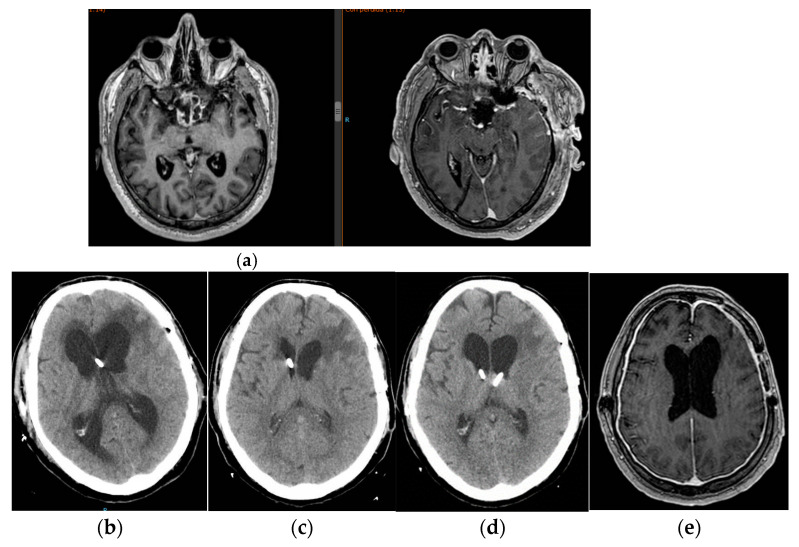
Image (**a**) pre-surgical and immediate post-surgical images of patient n 5 (**b**): development of hydrocephalus being treated with EVD. Image (**c**): normalization of ventricular size at day 25 after subzero drainage. Image (**d**): new ventricular dilatation despite definitive shunt treated by a new wide external catheter in left ventricle. Image (**e**): magnetic resonance imaging at discharge. Hyperdrainage signs are seen with ventricular size almost normalized.

It is noteworthy that the patient suffered a weight loss of approximately 30 kg during the process (up to 100 kg weight) factor that could facilitate the outflow of CSF. 5 years later no modifications to the shunt have been required (Figure 5).

### 3.6. Patient n. 6

16 years old. Male. The patient was admitted with severe brain trauma, requiring bifrontal craniectomy and VPS due to secondary hydrocephalus with chronic symptoms (gait and sphincter control impairment) despite the replacement of the cranial vault.

In the context of fever and obtundation, infection of the shunt system (programmable) was diagnosed 4 months later. VPS was externalized and internalized once the infection resolved. Nevertheless, he developed insufficiency of the said system.

He required EVD for several weeks with drainage at negative pressures not tolerating any increase in EVD above the EAC.

**Figure 6:**
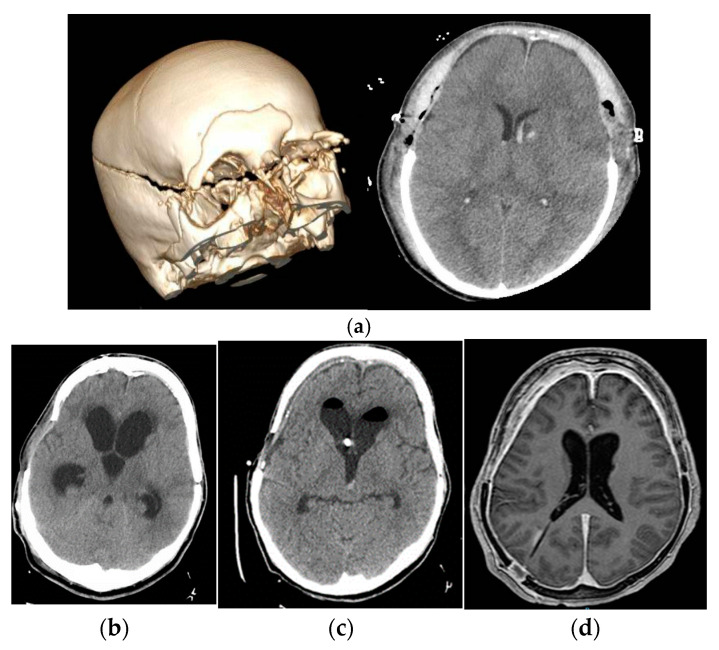
Image (**a**) First image at the patient admission and in the immediate postoperative period (**b**): development of hydrocephalus after the replacement of cranial vault, without clinical response with low-pressure shunt. Image (**c**): with EVD at negative pressures, the patient recovers their level of consciousness without tolerating any increase above the EAC. Image (**d**): shunt programmed at 9 mmHg with distal catheter in right atrium. Normalization of the level of consciousness and basal ventricular size with mild signs of hyper drainage in NMR, which suggests predominance of negative pressure exerted by the atrium.

After resolving CSF infections, in another center, he finally received a programmable ventricular atrial shunt (Codman CERTAS^®^), programmed in the range of 9 mmHg. However, radiological images after placement showed subtle signs of hyper drainage similar to those he had presented with the EVD below the EAC. We inferred that he continued to require sub-atmospheric pressures to maintain basal ventricular size, achieved by a balance between the negative pressure of the atrium and that established by the programmable shunt (Figure 6). The total time of forced negative pressure was 40 days.

## 4. Discussion

Since the first description of low-pressure hydrocephalus in 1994, approximately 200 cases have been reported in publications, grouped in small series. This leads us to conclude that this is an under-described situation (Table 2) [7,8,9,10,11,12,13].

Symptoms are comparable to those of classic hydrocephalus with intracranial hypertension.

Although several explanations have been proposed, its exact pathophysiology remains unknown. Pang and Altschuler initially used viscoelastic principles to explain this state: in the classical model of elasticity, changes in linear strain are observed in response to external forces, as described by Hooke’s law (strain proportional to stress). Stress applied over a long period of time may “soften” matter, which will behave like a viscous liquid.

Lesniak et al. [2] contribute to this theory and postulate that intermittent increases in pressure (intermittent shunt dysfunction) eventually lead to a loss of ventricular elastance that persists despite the cessation of high pressure (hysteresis). Negative pressures would be necessary to restore elastance. The ventricular system would behave in a similar way to the lung during inspiration and expiration: the intrapleural pressure is lower during expiration and higher during inspiration. This phenomenon has been observed in other organs such as the vocal cords, aorta, lungs, and cardiac muscle. These changes may be analogous to those occurring in the senile brain in normal-pressure hydrocephalus [2].

This theory is supported by several studies on brain elastance measured by magnetic resonance elastography in patients diagnosed with normotensive hydrocephalus treated with shunts [4,12].

Complementing viscoelastic theory is the poroelastic theory, which attributes the increase in ventricular size to an increase in extracellular permeability, which saturates the periventricular white matter and causes the ventricular wall to lose its elasticity. Forced drainage of the aqueous component would decrease this periventricular saturation and restore the ventricular wall to its original firmness and shape [14,15,16]. Other authors [8,13] predict a proportional association between periventricular oedema and the development of this type of hydrocephalus. They hypothesize that inflammatory or degenerative changes alter periventricular cerebral permeability [17,18,19].

Conditions responsible for this syndrome include long-time shunt carriers, subarachnoid or intraventricular hemorrhage, meningitis, tumors, or diffuse brain parenchymal damage. Particular susceptibility has been detected in posterior fossa pathologies, such as medulloblastomas and ependymomas. Finally, craniotomy or craniectomy can increase compliance and decrease the stiffness of the cranial vault, changing the relationship between pressure and brain volume.

In short, any damage to the parenchyma, CSF flow, or cranial vault may cause marked dilatation of the ventricular system with ventriculomegaly and paradoxically low pressure. It is, therefore, assumed that as long as there is ventricular dilatation, there is neuronal dysfunction due to the distraction of the white matter tracts.

In 2008, Rekate et al. [20] highlighted the important contribution to hydrocephalus of the cortical subarachnoid space (CSAS)—largely ignored so far—and considered the concept of communicating hydrocephalus to be archaic. They recommended insisting on the search for obstruction, for which multiple tools are available. They experimented with calculations of cranial, ventricular, and subarachnoid space volume based on measurements of the skull as a sphere. An almost constant differential pressure of 5–7 mmHg between the CSAS and the superior sagittal venous sinus is among their results. They consider the term “compliance” inappropriate and use brain “turgor” instead, stating that it can be rapidly disturbed not only by blood flow, but also by age, ischemia, external damage, or radiotherapy. This lack of turgor makes it impossible for CSF to flow into the ventricles. Therefore, the apparently contradictory maneuver of wrapping the patient’s neck would increase venous sinus pressure and distend the CSAS, thus encouraging CSF to pass between these compartments. They describe four cases resolved by this technique together with drainage at negative pressures and ventriculostomy of the third ventricle (ETV). Rekate et al. were able to document a block at some point of CSF flow measured by dynamic radiological studies after intrathecal contrast injection. None of the patients had a history of shunt implantation in infancy. These authors recommend extensive work-up, leak repair, and ETV to communicate the CSAS and the ventricular system in all cases where this possibility is available. Only a terminal problem in absorption can be considered communicating hydrocephalus.

Three years later, members of this same team published a series of three new cases [17], postulating that the cause of low-pressure hydrocephalus required the search for an occult CSF leak (especially in patients with a history of cranial base surgery), or other circumstances (such as hemorrhage or infection) producing a “disconnection” between the ventricles and the subarachnoid space. The leak would produce a vacuum effect, draining the subarachnoid space and causing ventricular dilatation, which, in turn, would block drainage from the ventricles into the subarachnoid space. Rekate et al. noted that this maneuver is more effective in younger subjects. They reported CSF obstruction at the occipitocervical junction after posterior fossa aneurysm surgery, with the help of radioactive isotope scans injected through the EVD. They resolved the obstruction by surgical repair of adhesions and distal catheter to the right atrium in one case. They argued that the keys are brain turgor and the pressure gradient between ventricles and subarachnoid space. All these actions were accompanied by somewhat prolonged treatment with external drainage at subatmospheric pressures.

Other authors had already observed the association between lumbar puncture and the development of very low-pressure hydrocephalus [21,22], postulating that ventricular size increased after puncture due to CSF outflow from the subarachnoid space only—not from the ventricles. In their series published in 2017, drawn entirely from pediatric cases—the longest to date (29 patients)—Smalley et al. [23] clearly differentiate between those patients who develop very-low-pressure hydrocephalus post-lumbar-puncture and those who do not.

Shortly after the formulation of these theories, two very interesting papers were published comparing results with and without ETV in their series:

1. In 2012, Hamilton and Price [24] treated 10 of their 20 patients with low-pressure hydrocephalus with ETV, combined with draining with a subzero system and with neck wrapping for some of them. All had signs of CSF obstruction, as shown in Cine NMR sequences. The etiologies were varied (post-meningitis, post-lumbar-puncture, intraventricular tumors, etc.). The authors found that in the group treated with ETV, 20% of the patients required a definitive shunt, whereas in the group without it, it was 70%. The authors support ETV, although they recognize that placing a shunt is faster and reduces hospital stay. They consider the use of shunts for cavities with negative pressures (pleura, heart).
2. Foster et al. (2016) [25] postulated that ETV could shorten the time of subzero drainage. In their series of 16 patients, 6 of them underwent ETV; 1 patient did not require a definitive shunt. The range of time until the basal ventricular size was achieved with negative drainage was from 1 to 236 days. They found no significant differences in the time of treatment between the two groups. The causes of hydrocephalus were diverse. The use of ETV depended on the preferences of the surgeon and the institution. Those patients with more favorable characteristics (no previous shunt carriers, nor hydrocephalus secondary to hemorrhage or meningitis) were selected for ETV. The authors concluded that it is difficult to predict who would benefit and who would not. The study is subject to the very heterogeneous characteristics of each patient (age, underlying pathology, etc.).

**Table 2:** Summary of published series.

Year	Authors	Population	Number	Technique
**1994**	Pang and Altschuler [3]	Children	12	Subzero drainage and peritoneal low pressure shunts.
**1999**	Dias et al. [22]	Children	2	Lumbar blood patch.
**2001**	Owler et al. [10]	Adults and children	5	Subzero drainage. Three peritoneal shunts valveless, one mild-pressure.
**2002**	Lesniak et al. [2]	Adults	10	Subzero drainage and low-pressure shunts.
**2006**	Clarke [11]	Adults	2	Subzero drainage. One vetriculo-atrial shunt and one peritoneal valveless shunt.
**2011**	P Akins et al. [9]	Adults and children	9	Subzero drainage and low-pressure shunts. Two pleural shunts.
**2011**	Filippidis [18]	Adults	3	Subzero drainage, neck wrapping, and CSF leak repair.
**2012**	Hamilton and Price [25]	Adults	20	Subzero drainage, neck wrapping, and ETV.
**2017**	Smalley [24]	Children	29	Lumbar blood patch, subzero drainage, and programmable shunts (one to pleura).
**2020**	Keough et al. [4]	Adults and children	42	Subzero drainage +/− ETV.
**2022**	Casado-Pellejero et al. [12]	Children	5	Early ETV, low pressure shunts
**2023**	Present series	Adults and children	6	Subzero drainage and programable shunts, one to right atrium.

The last published series on low-pressure hydrocephalus [24], which has already been discussed [23], is also the largest—with 29 pediatric cases. Following the above-described theory of Rekate’s team, Smalley et al. [23] used spinal hematic patches, with good outcomes in 20% of the patients. In one case, they performed an ETV, this being the only case in the series not resolved with a definitive shunt. Most of the cases involved tumor and hemorrhagic pathology. A novelty was the high rate of patients with digestive symptoms consisting of nausea and vomiting in half of the cases, as well as other symptoms not classically described, such as bradycardia. Negative pressure drainage had to be used in practically all cases.

## 5. Conclusions

With the current experience, it can be affirmed that this condition can develop in any context and in a manner comparable to the development of classic hydrocephalus, be it a tumor, after hemorrhages, malformations, after meningitis, or even as a progression of chronic adult hydrocephalus. Its form of presentation is clinically indistinguishable from high-pressure hydrocephalus and the absence of treatment also implies a risk of death, as well as severe neurological damage that may be permanent.

In our series, we have found (as described) the symptomatology of vomiting in the foreground in the case of two pediatric patients affected by medulloblastom.

This is the first description of this condition related to leptomeningeal glioneuronal tumor.

The recordings of continuous ICP in our patients with ventriculomegaly—in spite of low-pressure shunt systems or “free tubes”—do not show pathological waves, as observed in normotensive hydrocephalus patients. Signs of parenchymal stiffness are not present either, showing minimal differences between systolic and diastolic intracranial pressures.

According to our review, the “subzero” drainage method still seems to be the most effective and the most used, whether or not assisted by the other procedures already detailed.

The duration of treatment may vary and can last up to months depending on the published series. The search for available large-bore catheters is encouraged, since it can reduce the duration of treatment.

Shunt systems inside cavities with negative pressure or without valves can be useful as alternatives to external ventricular drainage.

The role of ETV is increasingly gaining attention, although there are still few cases that demonstrate its usefulness, especially in the most acute phase.

## Figures and Tables

**Table 1:** Patients diagnosed with low-pressure hydrocephalus during 2015–2020 period and details of their management.

Pat	Sex	Age	Initial Diagnosis	Time of Subzero Drainage	Final Shunt	Ranking Modified Score at 1 Year of Follow-Up
1	M	11	Medulloblastoma	---	Miethke, ProGav 2.0^®^ (*Potsdam, Germany*) adjusted in 0 mmHg	1
2	M	11	Medulloblastoma	10 days	Miethke ProSA^®^ (gravitational unit) nAdjusted in 0 mmHg	3
3	M	9	Diffuse leptomeningeal glioneuronal tumor	15 days	Miethke Progav 2.0 + ProSA adjusted in 0 mmHg	5
4	F	80	Adult Chronic Hydrocephalus	15 days	Miethke M-Blue^®^ (gravitational unit) adjusted in 0 mmHg,	2
5	M	43	Craniopharyngioma	35 days	Miethke ProSA^®^ adjusted in 0 mmHg	3
6	M	16	Severe brain Trauma. Descompressive Craniectomy	40 days	Codman Certas to rigth atrium adjusted in 9 mmHg	3

## Data Availability

The data presented in this study are available on request from the corresponding author.

## Abbreviations

CSF (cerebrospinal fluid), EVD (external ventricular drainage), VPS (ventriculoperitoneal shunt), ICP (intracranial pressure), ETV (endoscopic third ventriculostomy), EAC (external auditory canal), IH (intracranial hypertension), IIH (idiopathic intracranial hypertension), NMR (nuclear magnetic resonance), CT (computed tomography), CSAS (cortical subarachnoid space), LP (lumbar puncture).
