# Correction: Godoy Hurtado et al. Low- and Negative-Pressure Hydrocephalus: New Report of Six Cases and Literature Review. *J. Clin. Med.* 2023, *12*, 4112

**DOI:** 10.3390/jcm14030959

**Published:** 2025-02-02

**Authors:** Alicia Godoy Hurtado, Patrick Barstchi, Juan Francisco Brea Salvago, Rajab Al-Ghanem, Jose Manuel Galicia Bulnes, Osamah El Rubaidi

**Affiliations:** 1 Department of Neurosurgery, Jaén Neurotrauma Hospital, 23001 Jaen, Spain; 2 Department of Neurocritical Care, Jaén Neurotrauma Hospital, 23001 Jaen, Spain

Following publication, concerns were raised regarding the peer-review process related to the publication of this article [1]. Adhering to our standard procedure, the Editorial Office and Editorial Board investigated and determined that the contribution of one of the two reviewers did not comply with MDPI’s Guideline for Reviewers (https://www.mdpi.com/reviewers#_bookmark11 (accessed on 19 Jan 2024)), nor the expectations of the Editorial Board relating to the quality of the peer-review report. As a result, the Editorial Board decided to conduct a post-publication peer-review process, which included the recruitment of a new reviewer and re-evaluation by the original Academic Editor, to ensure that this publication fully complies with MDPI’s editorial process policy (https://www.mdpi.com/editorial_process (accessed on 19 Jan 2024)).

This process has now been completed and has resulted in a number of updates to the original publication (listed below), the removal of the linked Journal Notice, the removal of the original “reviewer 1” report, and the addition of the new report provided by the new reviewer on the open peer-review page connected to the article (https://www.mdpi.com/2077-0383/12/12/4112/review_report (accessed on 19 Jan 2024)). This correction was approved by the Academic Editor.

## Affiliation

In the published publication, the authors revised the postcode in affiliations 1 and 2, The corrected affiliations are as follows:

1. Department of Neurosurgery, Jaén Neurotrauma Hospital, 23001 Jaen, Spain
2. Department of Neurocritical Care, Jaén Neurotrauma Hospital, 23001 Jaen, Spain

## Text Correction

There were some errors in the original publication. A correction has been made to “Abstract”; the authors revised a sentence “a patient carrier a shunt for normotensive hydrocephalus diagnosed ten years before. At the moment of development”.

A correction has been made to “Keywords”; the authors added a new Keyword “leptomeningeal glioneural tumor”.

A correction has been made to “Section 1 Paragraph 2”; the authors revised a word from cronic to chronic, and revised Hydrocepahlus to Hydrocephalus.

A correction has been made to “Section 2 Paragraph 1”; the authors revised a word from hydrocepahlus to hydrocephalus. A correction has been made to “Section 2 Paragraph 5”; the authors added a “some” in a sentence. A correction has been made to “Section 2 Paragraph 7”; the authors revised “was” to “lasted”.

A correction has been made to “Section 3 Paragraph 1”; the authors revised paragraph 1 from “The most important aspects are summarized in Table 1.” to “Bellow we develop the details of their evolution (Table 1).”.

A correction has been made to “Section 3”; the authors deleted the paragraphs 2–6 and added a new Section 3.1:

### 3.1. Patient n. 1

11 year-old male patient was diagnosed with a posterior fossa lesion after consulting for vomiting and one month duration progressive headache and strabismus. Surgery was performed and complete resecction was achieved with histological resulting in desmoblastic medulloblastoma without meningeal involvement. 15 days after surgery the patient needed a CSF shunt due to clinical stagnation and increased ventricular size. Low pressure shunt was placed (Gav 5/35 Miethke^®^) with standardization of ventricular size, nevertheless the patient was affected by akinetic mutism and significant prostration. Radio and chemotherapy schedule was administered during following 2 months. The NMR after treatment showed considerable increase in venticular size so it was decided to replace shunt with Medtronic, Strata^®^ programmable system (Minneapolis, MN, USA) adjusted in 0.5. (1.5 mmHg). At this point the patient showed severe neurological impairment, without contact with the environment. In the absence of clinical or radiological response, multiple surgical revisions were carried out, including externalization of catheter several times.

Finally, the patient received an antigravitational device (Miethke, ProGav 2.0^®^ (Potsdam, Germany) associated with shunt and adjusted in 0 mmHg. Clinical and radiological improvement start one week after surgery. The patient could be discharged three weeks later, being able to recover oral feeding for the first time after the first surgery.

Overall hospitalization lasted 5 months since the first shunt dysfunction. Recovery continued until reaching a 80% Karnofsky Performance status 4 months later.

Opening pressure reprogramming was needed to 4 mmHg due to asymptomatic subdural collections. 5 years after placement it maintains the same shunt system and opening pressure (Figure 1).

The authors revised Section “3.1.1” to Section “3.2”.

The authors revised Section “3.1.2” to “3.3”. Correction has been made to “Section 3.3 Paragraph 3”. The authors revised the System version from “(Codman Certas^®^)” to “(Codman Certas Plus^®^)”.

The authors revised Section “3.1.3” to “3.4”.

Correction has been made to “Section 3.4 Paragraph 1”: The Authors revised the first three sentences to the following: 80 years old. Female. The patient was diagnosed ten years earlier with chronic adult hydrocephalus in another center after consulting for gait disturbance. She received low-pressure VPS with an antigravitational unit with good response during this time.

Correction has been made to “Section 3.4 Paragraph 2”: The authors added a sentence “The whole time with forced negative pressure was 15 days” to this paragraph.

Correction have been made to “Section 3.4 Paragraph 3”: The authors added a sentence “The patient was asymptomatic.”

The authors added a Section 3.5, the corrected paragraph:

### 3.5. Patient n. 5

42 years old. Male. History of retroperitoneal fibrosis.

The patient was diagnosed with craniopharyngioma after consulting for visual loss. He underwent surgery through a left pterional approach. The postoperative period was complicated with panhypopituitarism and septicemia of urinary origin. At 7° day after surgery he was diagnosed with hydrocephalus by CT. Clinically showed sleep tendency.

A programmable shunt system was placed (Codman CERTAS plus^®^) with progressive pressure drop at lowest level without clinically or radiologically improvement. Surgical revisions and catheter externalizations were carried out ineffectively. The patient responded to EVD draining at negative pressures which were reached progressively to 15 cm below de EAC, with daily debts around 300 mL of CSF. It took 25 days at intensive unit care to recover normal ventricular size associated with level of consciousness maintained. Then level of EVD was progressively raised until Monro level. On day 40 after the onset, he could receive a new shunt associated with an antigravitational device programmed at zero opening pressure with success (Miethke ProSA^®^). 7 days later the patient presented low level of consciousness again with a new ventricular dilatation. At this moment it was decided to place a concomitant catheter in contralateral ventricle and start again negative drainage. For 20 days he was maintained with the double catheter system. Progressive removal of the external catheter was possible as done previously.

It is noteworthy that the patient suffered a weight loss of approximately 30 kg during the process (up to 100 kg weight) factor that could facilitate the outflow of CSF. 5 years later no modifications to the shunt have been required (Figure 5).

The authors revised Section “3.1.4” to “3.6”. Corrections have been made to “Section 3.6 Paragraph 1”; the authors revised the first paragraph.

Corrections have been made to “Section 3.6 Paragraph 4” and “Section 5 Paragraph 2”; the authors add some new words in the paragraph.

The authors added a paragraph to “Section 5 Paragraph 3”:

This is the first description of this condition related to leptomeningeal glioneuronal tumor.

## Error in Figure/Table

There was a mistake in Table 1 as published. The author deleted the fifth and sixth columns. The corrected Table 1 appears below.

**Table 1:** Patients diagnosed with low-pressure hydrocephalus during 2015–2020 period and details of their management.

Pat	Sex	Age	Initial Diagnosis	Time of Subzero Drainage	Final Shunt	Ranking Modified Score at 1 Year of Follow-Up
1	M	11	Medulloblastoma	---	Miethke, ProGav 2.0^®^ (*Potsdam, Germany*) adjusted in 0 mmHg	1
2	M	11	Medulloblastoma	10 days	Miethke ProSA^®^ (gravitational unit) nAdjusted in 0 mmHg	3
3	M	9	Diffuse leptomeningeal glioneuronal tumor	15 days	Miethke Progav 2.0 + ProSA adjusted in 0 mmHg	5
4	F	80	Adult Chronic Hydrocephalus	15 days	Miethke M-Blue^®^ (gravitational unit) adjusted in 0 mmHg,	2
5	M	43	Craniopharyngioma	35 days	Miethke ProSA^®^ adjusted in 0 mmHg	3
6	M	16	Severe brain Trauma. Descompressive Craniectomy	40 days	Codman Certas to rigth atrium adjusted in 9 mmHg	3

The authors added Figure 1 in Section 3.1:

**Figure 1:**
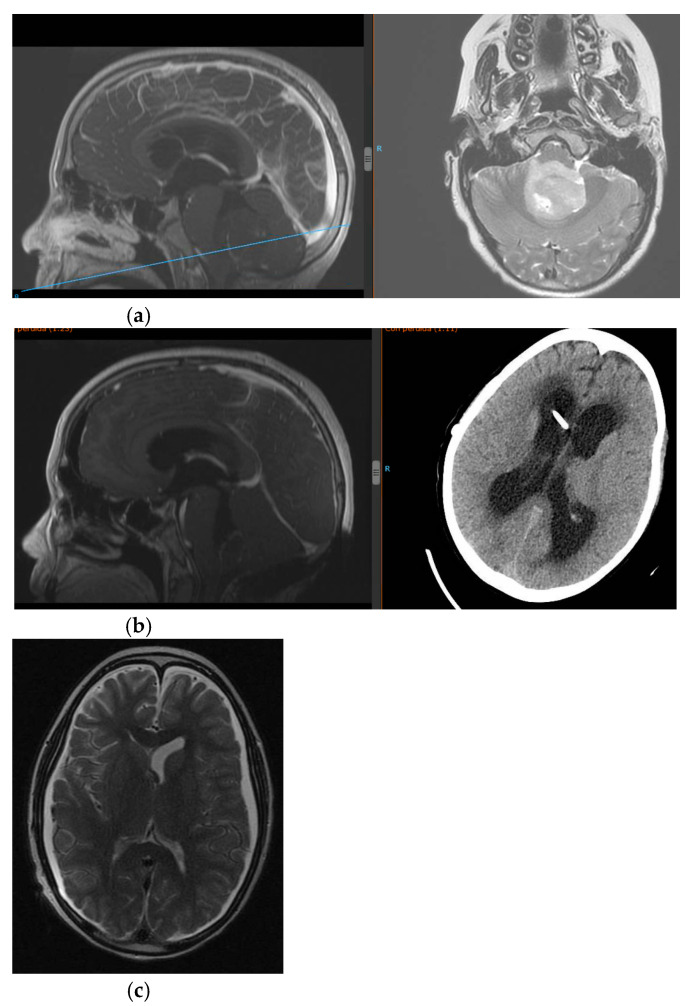
(**a**) NMR at admission showing fourth ventricle lesion and obstructive hydrocepahlus. (**b**) Postsurgical period with development of low-pressure hydrocephalus, needing several shunts revisions. (**c**) NMR one month after the placement of shunt asociated a PROSA^®^ device as “a free tube”.

The authors added Figure 5 in Section 3.5:

**Figure 5:**
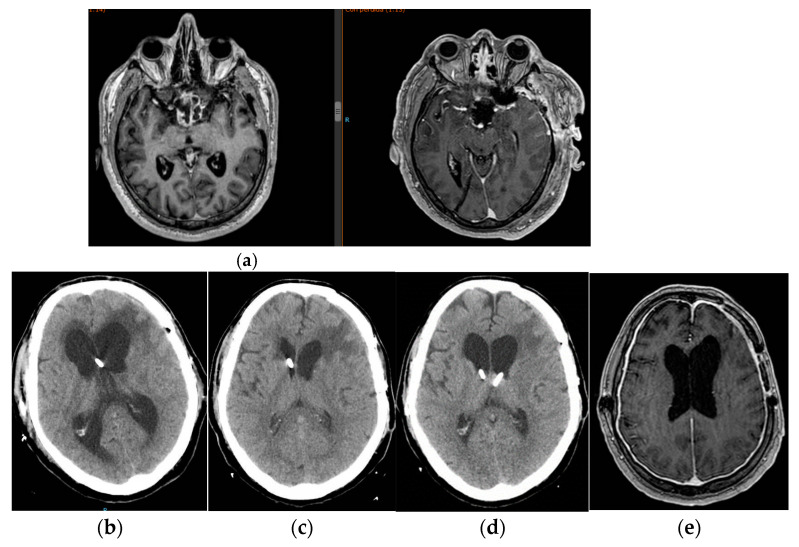
Image (**a**) pre-surgical and immediate post-surgical images of patient n 5 (**b**): development of hydrocephalus being treated with EVD. Image (**c**): normalization of ventricular size at day 25 after subzero drainage. Image (**d**): new ventricular dilatation despite definitive shunt treated by a new wide external catheter in left ventricle. Image (**e**): magnetic resonance imaging at discharge. Hyperdrainage signs are seen with ventricular size almost normalized.

The authors revised Figure 6 in Section 3.6:

**Figure 6:**
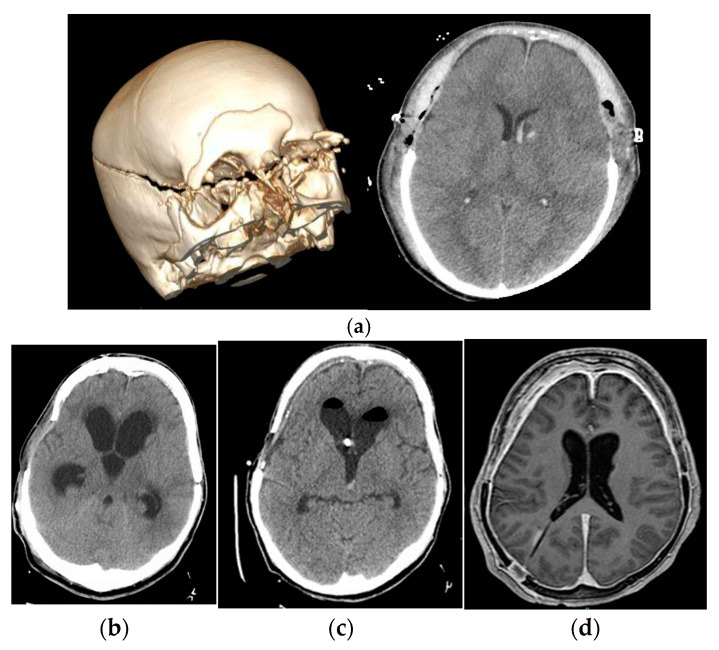
Image (**a**) First image at the patient admission and in the immediate postoperative period (**b**): development of hydrocephalus after the replacement of cranial vault, without clinical response with low-pressure shunt. Image (**c**): with EVD at negative pressures, the patient recovers their level of consciousness without tolerating any increase above the EAC. Image (**d**): shunt programmed at 9 mmHg with distal catheter in right atrium. Normalization of the level of consciousness and basal ventricular size with mild signs of hyper drainage in NMR, which suggests predominance of negative pressure exerted by the atrium.

With this correction, the order of some figures has been adjusted accordingly. The authors state that the scientific conclusions are unaffected. This correction was approved by the Academic Editor. The original publication has also been updated.
